# Clinical Outcomes and Provoked Epicardial Spasm Phenotypes via Intracoronary Acetylcholine Testing in 680 Patients with Angina and Nonobstructive Coronary Arteries

**DOI:** 10.3390/life12101465

**Published:** 2022-09-21

**Authors:** Shozo Sueda, Keisho Kurokawa, Tsukasa Kurokawa, Tomoki Sakaue, Shuntaro Ikeda

**Affiliations:** 1 Department of Cardiology, Ehime University, Ehime 790-8577, Japan; 2 The Department of Cardiology, Ehime Prefectual Niihama Hospital, Hongou 3 Choume 1-1, Niihama 792-0042, Japan

**Keywords:** combined spasm, diffuse spasm, epicardial spasm, focal spasm, nonobstructive coronary artery disease, prognosis

## Abstract

**Background**: Epicardial spasm (ES) phenotypes may be related to the prognosis in patients with coronary spastic angina. **Objectives**: The purpose of this study was to elucidate the relationship between angiographic coronary vasomotor responses to intracoronary acetylcholine (ACh) injection and prognosis in patients with angina and nonobstructive coronary artery disease (ANOCAD). **Methods**: This was a retrospective, observational, single-center study of 680 patients with ANOCAD. ACh spasm provocation tests on both coronary arteries were performed without administering nitroglycerine to relieve provoked spasm in a first-attempt artery. ACh was injected in incremental doses of 20/50/100/200 μg into the left coronary artery and 20/50/80 μg into the right coronary artery. Positive ES was defined as ≥90% stenosis and usual chest pain and ischemic ECG changes. **Results**: Provoked positive ES was observed in 310 patients (46%), including 85 patients (13%) with focal spasm, 150 patients (22%) with diffuse spasm, and 75 patients (11%) with combined spasm (diffuse spasm and focal spasm), whereas the remaining 370 patients (54%) had no provoked spasm. An unclassified ACh test was observed in 186 patients (27%), while 184 patients (27%) had a complete negative ACh test. The clinical outcomes in patients with complete negative ES were satisfactory compared with those with positive ES and unclassified ACh test results. The prognosis in patients with an unclassified ACh test was not different from those with a positive ES. Furthermore, prognosis in patients with ES phenotypes was not different among the three groups. **Conclusions**: There was no correlation between provoked ES phenotypes via intracoronary ACh testing and prognosis in patients with ANOCAD; however, clinical outcomes in patients with positive ES and unclassified ACh tests were worse compared to those with complete negative ACh tests. We should focus on the treatments in patients with unclassified ACh tests as well as those with ESs.

## 1. Introduction

Approximately one half of patients undergoing diagnostic coronary angiography for typical chest pain have no significant coronary artery stenosis [1]. In these patients with angina and nonobstructive coronary artery disease (ANOCAD), coronary functional abnormalities, including epicardial spasm (ES), coronary microvascular spasm (CMS), or coronary microvascular dysfunction, could be involved [2]. Vasoreactivity testing as well as coronary physiological functional testing using a guidewire are the pivotal methods. In 1986, intracoronary acetylcholine (ACh) testing was first reported [3]. Since then, the intracoronary ACh test and the ergonovine (ER) test have become popular as vasoreactivity tests [4,5,6,7,8]. Coronary artery spasm may concern an acute coronary syndrome, unstable angina, sudden cardiac death, serious fatal arrhythmia, cryptogenic syncope, or an unknown origin of heart failure [9,10,11,12]. According to previous studies, physicians described two patterns of provoked spasm: focal and diffuse spasm. Although there is no responsibility attributed to ACh injection, physicians defined provoked spasm as focal or diffuse in a one-point diagnosis in the cardiac catheterization laboratory. However, whether the inducible spasm phenotypes are related to the prognosis is controversial. In this article, we investigated the correlation between angiographic manifestations of provoked spasm via intracoronary injection of ACh and long-term clinical outcomes of patients with ANOCAD who underwent intracoronary ACh testing on both coronary arteries.

## 2. Methods

### 2.1. Study Patients

**Figure 1:**
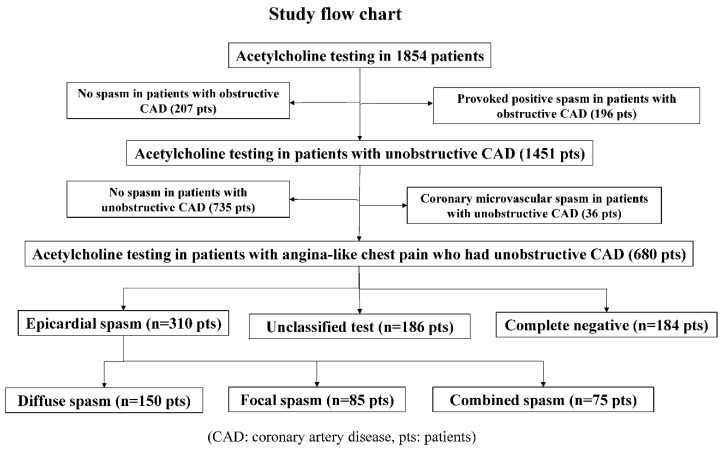
Study flow chart.

From January 1991 to February 2019, we performed a total of 8351 coronary angiography procedures, including 2353 percutaneous coronary intervention procedures and 5998 diagnostic and follow-up cardiac catheterization procedures. During the same period, we performed ACh spasm provocation tests in 1854 patients, and we performed selective spasm provocation tests to examine the incidence of provoked spasm in patients who had undergone coronary angiography whenever possible. As shown in Figure 1, we enrolled 680 patients who had ANOCAD. We excluded 36 patients with CMSs, including 4 patients with ESs.

### 2.2. Definition of Positive Spasm, Phenotypes of Provoked Spasm, and Major Cardiac Adverse Events

We defined positive ES as ≥90% transient narrowing and usual chest symptoms and ischemic ECG changes [13,14]. We also defined positive CMS as <75% transient narrowing (no epicardial spasm) and usual chest symptoms and ischemic ECG changes. Patients who experienced no angina, no ES or CMS, and no ST-segment shifts were considered to have a complete negative ACh test response. Patients who had either one or two issues but not all three issues (>90% transient narrowing, usual chest symptoms, or ischemic ECG changes) were defined as an unclassified ACh test. We defined negative ACh test as complete negative ACh test and unclassified ACh test. During and/or after the ACh test, we considered a result to be positive when there was ST-segment elevation or depression of ≥0.1 mV in at least two contiguous leads or negative U wave. Obstructive coronary artery disease was defined as ≥50 percent luminal narrowing, according to the American College of Cardiology (ACC)/American Heart Association (AHA) classification [15]. As defined by the AHA, coronary spasm is defined as a total or subtotal obstruction within the borders of one isolated coronary segment (focal spasm (FS)), or severe diffuse vasoconstriction (90% stenosis) observed in ≥2 adjacent coronary segments (diffuse spasm (DS)) of the epicardial coronary arteries, and associated with transient myocardial ischemia, as evidenced by ischemic ECG changes. In this study, we divided the patients who tested positive on the ACh spasm provocation test into three groups based on the coronary artery spasm characteristics observed on the coronary angiography during the ACh spasm provocation test; those with FS, those with DS, or those with combined spasm ((CS), focal spasm and diffuse spasm)). We also categorized major adverse cardiac events (MACEs) during follow-up periods as admission necessary for unstable angina, sudden cardiac death, ventricular fibrillation/tachycardia, heart failure, cerebral infraction, percutaneous coronary intervention, or acute coronary syndrome.

### 2.3. Spasm Provocation Test 

All drugs except for nitroglycerine were discontinued for ≥24 h before the study, and nitroglycerine was also discontinued ≥4 h before the study. Coronary angiography was performed in the fasting state. After control coronary arteriograms of both coronary arteries (right coronary artery (RCA) and left coronary artery (LCA)), we inserted a temporary pacemaker into the right ventricle and set the pacing rate at 40–45 beats/min. Provocation of coronary artery spasm was performed with an intracoronary injection of ACh, as previously reported [16,17]. ACh chloride was injected in incremental doses of 20/50/80 μg into the RCA and of 20/50/100/200 μg into the LCA over 20 s. Coronary angiography was performed when ischemic ECG changes and/or chest pain occurred 1–2 min after the completion of each ACh injection. When a documented coronary spasm did not resolve spontaneously within a couple of minutes or when hemodynamic instability occurred as the result of coronary spasm, 2.5–5.0 mg of nitrate was injected into the involved vessel. After the ACh vasoreactivity tests were completed, abundant isosorbide dinitrate (5.0 mg) was administered intracoronary, and coronary arteriography was performed in multiple projections. Patients with catheter-induced spasms were excluded from this study. 

The study protocol complied with the Declaration of Helsinki. Written informed consent was obtained from all patients before performing the ACh vasoreactivity tests, and the protocol of this study was consistent with the guidelines of the ethical committee at our institution.

### 2.4. Statistical Analysis

Data analysis was conducted with SPSS (version 22.0, IBM Japan, Ltd., Tokyo, Japan). All data are presented as the mean ±1 SD. Clinical characteristics, including coronary risk factors, provoked spasm incidence, medications, and cardiac events during the follow-up period, were analyzed by Fisher’s exact test with correction or the Mann–Whitney test. Multiple logistic regression was performed by using forward variable selection based on the likelihood ratios to identify predictors of a positive ACh test and negative ACh test. Event-free survival curves from MACEs were constructed using the Kaplan–Meier survival method. *p* < 0.05 was considered significant.

## 3. Results

### 3.1. Comparisons of Clinical Characteristics between ACh-Induced Positive Spasm and ACh-Negative Spasm

As shown in Table 1, of the 680 patients with ANOCAD, positive spasm by intracoronary ACh testing was observed in 310 patients (46%), while negative spasm was found in the remaining 370 patients (54%). The frequency of males, resting chest pain, history of smoking, and the use of calcium channel blockers (CCBs) and nitrates/nicorandil in patients with positive spasm were markedly higher than those in patients with negative spasms. “Another chest symptom” was markedly observed in more patients with negative spasms than in those with positive spasms.

### 3.2. Comparisons of Clinical Characteristics among Epicardial Spasm, Unclassified ACh Test, and Complete Negative ACh Test 

Table 2 shows that the incidence of males, resting chest pain, history of smoking, and use of CCBs/nitrates/nicorandil in patients with epicardial spasms were markedly higher than those in patients with unclassified ACh tests and complete negative ACh tests. “Another chest symptom” was significantly lower in patients with epicardial spasm than in the other groups. In epicardial spasms, RCA, left circumflex artery (LCX), and left anterior descending artery (LAD) spasms were observed in 266 patients, 119 patients, and 226 patients, respectively. Single-vessel spasms were revealed in 155 patients (50%), while multiple spasms were revealed in 155 patients (50%), including 79 patients (25.5%) with two-vessel spasms and 76 patients (24.5%) with triple-vessel spasms.

### 3.3. Clinical Comparisons Based on the Phenotypes of Provoked Spasm among the Three Groups

As shown in Table 3, DS was observed in 150 patients (48%), while 85 patients (27%) showed FS. The remaining 75 patients (24%) demonstrated CS. Fewer female patients had FS than DS. The follow-up duration in patients with FS was markedly lower than that in the other groups. The incidence of all three-vessel-provoked spasms in patients with DS was significantly higher than that in patients with FS. Furthermore, the incidence of all three-vessel spasms in patients with CS was markedly higher than that in patients with FS. Hypertension in patients with DS was higher than in those with FS and CS. More patients with FS had a history of smoking than those with DS. The use of statins was lower in patients with FS than in patients with other types of provoked spasms.

### 3.4. Kaplan–Meier Survival Curve and Multiple Logistic Analysis

**Figure 2:**
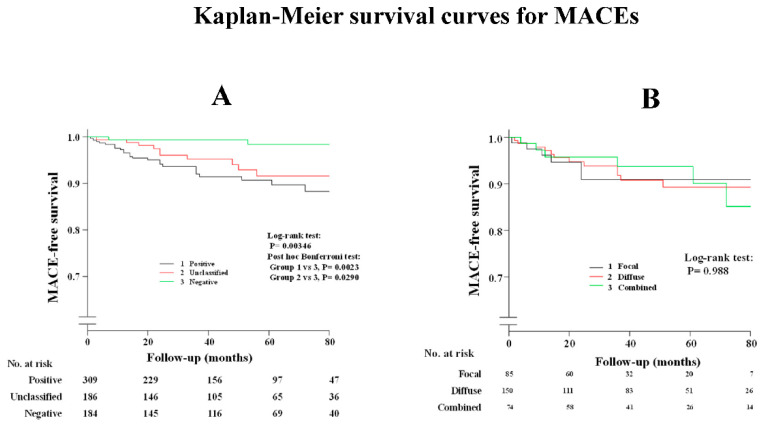
Kaplan–Meier survival curve for MACEs (**A**) in all study patients; (**B**) among provoked spasm phenotypes.

Supplementary Table S1 shows that a history of smoking and administration of CCBs and nitrates/nicorandil were the determinant factors after the multivariable analysis between patients with and without spasm. As shown in Supplementary Table S2, the administration of nitrates or nicorandil was the determinant factor after the multivariable analysis between patients with positive spasm and unclassified ACh test results. The occurrence of MACEs in patients with ES were the same for patients with unclassified ACh tests, as shown in Figure 2A and Table 4, while prognosis in patients with complete negative ACh tests was satisfactory when compared with the other two groups. Clinical outcomes were the same among the three provoked spasm phenotypes (DS/FS/CS), as shown in Figure 2B and Table 4. Readmission due to unstable angina pectoris in patients with DS was remarkably higher than that in those with negative ACh test, whereas there was no difference among the three provoked spasm phenotypes. Death due to malignancy was observed in three patients: one lung cancer, one pancreatic cancer, and one bile duct cancer. All three patients were found to have complete negative ACh results, while no malignancy was observed in the remaining groups during the follow-up periods.

### 3.5. Complications

We experienced 19 complications (3%), including 11 patients with nonsustained ventricular tachycardia, 1 with sustained ventricular tachycardia, 5 patients who experienced blood pressure drops (<60 mmHg), 1 patient with ventricular fibrillation, and 1 with left main trunk spasm equivalence. Provoked spasms were observed in 12 RCAs, while inducible spasms were observed in 7 LCAs. Electrical cardioversion was necessary for two patients; however, we had no irreversible complications, such as myocardial infarction or a requirement for cardiac resuscitation. All 680 patients who had intracoronary ACh testing were discharged the next day without any complications.

## 4. Discussion

In this article, we enrolled patients undergoing intracoronary ACh testing on both coronary arteries without the use of nitroglycerine to relieve provoked spasm in a first-attempt artery. All 680 study subjects were diagnosed by complete intracoronary ACh testing on both coronary arteries. We found no clinical prognostic impacts on the provoked spasm phenotypes (DS/FS/CS) in patients with ANOCAD. Furthermore, the clinical outcomes in patients with an unclassified ACh test were unfavorable, as was the prognosis in those with ES.

### 4.1. Comparisons of the Previous Reports

There are conflicting fact-based reports regarding clinical outcomes and provoked spasm phenotypes. Sato et al. reported that ACh-induced DS without severe coronary epicardial stenosis was associated with a better prognosis than FS [18]. They suggested that ACh-provoked coronary spasm subtypes in patients with coronary spastic angina (CSA) need to be identified. Furthermore, Nishiyama et al. also reported worse clinical outcomes in patients with ACh-induced FS compared with DS [19]. In contrast, recurrent chest pain was frequently observed in patients with DS compared with those with FS, while the 3-year clinical outcomes, including mortality, cardio and cerebrovascular disease, and recurrent chest pain, were similar between the focal and diffuse spasms in Korean populations [20]. However, the clinical outcome in this study was the same among the three groups: FS, DS, or CS types. Our study suggests that spasm-provoked phenotypes are not related to clinical outcomes. Sato et al. classified the two patterns of provoked spasm: DS, which was only ACh-induced DS, and FS, which was ACh-induced FS with or without DS. Nishiyama et al. investigated the provoked spasm phenotypes of left anterior descending artery alone but not all three coronary arteries. We classified our study populations into three groups based on inducible spasm phenotypes. Approximately half of the patients in Sato et al. and in Nishiyama et al. were females; however, less than 30% of our study subjects were females. The small number of female patients in our study groups, compared with the data of Sato et al. and Nishiyama et al., may have led to the difference in the results. In patients with ACh-inducible positive spasm, the incidence in females was markedly lower in our study than in Sato et al. (30% (65/216) vs. 44% (386/873), *p* < 0.001) and in Nishiyama et al. (30% (65/216) vs. 47% (115/246), *p* < 0.001).

### 4.2. Provoked Spasm Phenotypes by the Pharmacological Agents

ACh acts through muscarinic cholinergic receptors, while ergonovine acts by way of serotonergic receptors. Different mediators may have different coronary response between the two agents even in the same patients. According to our previous reports [21,22], the concordance of both (ACh and ergonovine) provoked spasm sites, and spasm configurations in the same coronary artery were observed in only 13% (18/134) of vessels. Diffuse and distal spasms were often observed by ACh testing, while focal and proximal spasms were often found by ergonovine tests in the same patients [21]. Furthermore, especially in the RCA of the same patients, intracoronary ACh injection provoked a distal spasm at segment 4, whereas intracoronary administration of ergonovine provoked a spasm at segment 2 [22]. This may be due to the distribution of cholinergic and serotonergic coronary receptors. Considering these results, provoked spasm phenotypes by vasoreactivity testing may be less meaningful regarding prognostic impact. According to the report of Akasaka et al. [23], coronary flow reserve was maintained in patients with FS compared with those with DS; however, we did not verify the difference in coronary flow reserve between patients with DS and FS. Age and duration of angina were the determinant factors for coronary flow reserve in patients with provoked spasm [24]. There was no difference in the coronary flow reserve regarding the provoked spasm phenotypes. Furthermore, intravascular ultrasound revealed diffuse intimal thickening in patients with DS, while FSs had some atheromatous plaque at the segmental area alone [25]. FSs have abnormal function at the limited segmental sections, whereas DSs have endothelial abnormalities throughout the entire coronary artery. The severity of coronary spasm in patients with DS may be a high-risk category compared with that in those with FS. In our previous report [26], DS revealed a refractory state compared with FS, irrespective of the use of coronary vasodilators. More importantly, cardiologists should accurately administer medications to VSA patients, irrespective of the appearance of provoked spasm subtypes.

### 4.3. Necessity of Optimal Management in Patients with Unclassified ACh Tests 

According to the report by Seitz et al. [27], the primary endpoints (all-case death and cardiac death) were the same among the four groups (epicardial spasm/microvascular spasm/normal ACh test result/inconclusive ACh test result). In contrast, secondary endpoints (nonfatal myocardial infarction/repeat coronary angiography/percutaneous coronary intervention/coronary aorta bypass grafting/stroke) in patients with ES (22.3% (55/247)) were higher than those in the other three groups (CMS: 13.5% (25/185), normal ACh test result: 12.9% (12/93), inconclusive ACh test result: 15.2% (32/211). In their study, the prognosis in patients with inconclusive ACh test results was not different from that in patients with normal ACh test results. Only 50% of their patients with ES were treated with CCBs at follow-up, possibly due to side effects. In contrast, in our study, the prognosis in patients with an unclassified ACh test was similar to that in those with ES. Furthermore, according to previous reports, patients with moderate vasomotor response (50–75%) or inconclusive ACh test (vasoconstriction and/or electrocardiographic changes, but no chest pain) had MACEs similar to those with ES [28,29]. Treating patients with moderate vasoconstriction and inconclusive vasoreactivity testing with optimal medications is challenging.

### 4.4. Limitations

We had several limitations in this study. First, the study was retrospective, single-center, and small. Second, we analyzed the medications before the pharmacological spasm provocation tests. We could not investigate the serial changes in medications in each patient during the follow-up periods. Third, intracoronary ACh injection of 80 μg into the RCA and 200 μg into the LCA was off label use according to the JCS guidelines. Fourth, the follow-up duration was too short to analyze the cardiac events among the four groups. Fifth, the use of statins and renin–angiotensin inhibitor/angiotensin receptor blockers in patients with focal spasm was remarkably lower than that in the other groups. The clinical outcomes were a concern because some researchers reported that the use of statins improved the clinical outcomes in patients with CSA [30]. Sixth, we could not analyze the spasm sites because we focused on the correlation between provoked spasm phenotypes and prognosis in this study. Provoked spasm sites via intracoronary ACh testing may be relevant to the prognosis. Further worldwide examinations are necessary to investigate the most clinically useful methods to provoke coronary spasm in the cardiac catheterization laboratory.

## 5. Conclusions

This study suggests that provoked spasm phenotypes via intracoronary ACh testing did not reflect prognosis in patients with CSA and nonobstructive coronary artery disease. Clinical outcomes in patients with ES and unclassified ACh tests were worse compared with those in patients with complete negative ACh tests. We should provide optimal pharmacological treatments to patients with unclassified ACh tests as well as those with positive spasms.

## Figures and Tables

**Table 1:** All patient clinical characteristics.

	All Patients	ACh Definite Positive	ACh Negative	*p* Value
Number	680	310	370	
Sex (female)	216 (32)	64 (21)	152 (41)	<0.001
Age, year, mean ± SD	64 ± 11	64 ± 10	64 ± 11	0.9907
Follow-up duration month, mean ± SD	50 ± 32	48 ± 31	52 ± 32	0.9907
Type of chest symptom				
Resting chest pain	396 (58)	228 (74)	168 (45)	<0.001
Exertional chest pain	76 (11)	31 (10)	45 (12)	0.3727
Effort and resting chest pain	84 (12)	43 (14)	41 (11)	0.2708
Another chest symptom	124 (18)	8 (3)	116 (31)	<0.001
ACh spasm testing				
Both coronary	680 (100)	310 (100)	370 (100)	
LVEF by UCG (%) mean ± SD	67 ± 8	67 ± 9	68 ± 7	0.7885
Coronary risk factors				
Hypertension	265 (39)	116 (37)	149 (40)	0.4477
Dyslipidemia	306 (45)	151 (49)	155 (42)	0.0751
Diabetes mellitus	134 (20)	55 (18)	79 (21)	0.2386
History of smoking	445 (65)	242 (78)	203 (55)	<0.001
Medications before ACh testing				
Calcium channel blocker	355 (52)	207 (67)	148 (40)	<0.001
Nitrate or nicorandil	266 (39)	168 (54)	98 (26)	<0.001
Beta blocker	44 (6)	15 (5)	29 (8)	0.1133
ACEI or ARB	97 (14)	44 (14)	53 (14)	0.9612
Statin	126 (19)	66 (21)	60 (16)	0.0898

(ACh: acetylcholine, LVEF: left ventricular ejection fraction, UCG: ultrasound cardiography, ARB: angiotensin receptor blocker, ACEI: angiotensin converting enzyme inhibitor).

**Table 2:** Patient clinical characteristics among three groups.

	Epicardial Spasm	Unclassified ACh Test	ACh Complete Negative
Number	310	186	184
Sex (female)	65 (21)	58 (31) *	95 (52) ***###
Age, year, mean ± SD	64 ± 10	64 ± 12	64 ± 10
Follow-up duration, month, mean ± SD	48 ± 31	50 ± 33	53 ± 32
Type of chest symptom			
Resting chest pain	229 (74)	107 (58) ***	60 (33) ***###
Exertional chest pain	31 (10)	24 (13)	22 (12)
Effort and resting chest pain	42 (14)	28 (15)	12 (7) *##
Another chest symptom	8 (3)	27 (15) ***	90 (49) ***###
ACh spasm testing			
Both coronary artery	310 (100)	186 (100)	184 (100)
RCA spasm	266 (86)	0	0
LCX spasm	119 (38)	0	0
LAD spasm	226 (73)	0	0
1 vessel epicardial spasm	155 (50)	0	0
2 vessel epicardial spasm	79 (25)	0	0
3 vessel epicardial spasm	76 (25)	0	0
LVEF by UCG (%) mean ± SD	67 ± 9	68 ± 8	67 ± 7
Coronary risk factors			
Hypertension	116 (37)	72 (39)	78 (42)
Dyslipidemia	150 (48)	87 (47)	68 (37) *
Diabetes mellitus	55 (18)	41 (22)	37 (20)
History of smoking	240 (77)	124 (67) **	78 (42) **###
Medications before ACh testing			
Calcium channel blocker	207 (67)	98 (53) **	52 (28) ***###
Nitrate or nicorandil	167 (54)	65 (35) ***	40 (22) ***##
Beta blocker	15 (5)	11 (6)	18 (10) ***
ACEI or ARB	44 (14)	14 (8) *	27 (15) #
Statin	64 (21)	33 (18)	25 (14) *

(ACh: acetylcholine, RCA: right coronary artery, LCX: left circumflex artery, LAD: left anterior descending artery, LVEF: left ventricular ejection fraction, UCG: ultrasound cardiography, ARB: angiotensin receptor blocker, ACEI: angiotensin converting enzyme inhibitor, *: *p* < 0.05, **: *p* < 0.01, ***: *p* < 0.001 vs. epicardial spasm, #: *p* < 0.05, ##: *p* < 0.01, ###: *p* < 0.001 vs. unclassified ACh test).

**Table 3:** Patient clinical characteristics according to the spasm phenotypes.

	Diffuse Spasm	Focal Spasm	Combined Spasm
Number	150	85	75
Sex (female)	44 (29)	7 (8) ***	13 (17)
Age, year, mean ± SD	65 ± 10	65 ± 9	63 ± 11
Follow-up duration, month, mean ± SD	51 ± 32	38 ± 27 **###	54 ± 33
Type of chest symptom			
Resting chest pain	109 (73)	64 (75)	55 (73)
Exertional chest pain	20 (13)	5 (6)	6 (8)
Effort and resting chest pain	16 (11)	14 (16)	13 (17)
Another chest symptom	5 (3)	2 (2)	1 (1)
ACh spasm testing			
Both coronary artery	150 (100)	85 (100)	75 (100)
RCA spasm	131 (87)	63 (74) *	72 (96) ###
LCX spasm	62 (41)	10 (12) ***	47 (63) **###
LAD spasm	122 (81)	36 (42) ***	68 (91) ###
1 vessel epicardial spasm	74 (49)	56 (66) *	21 (28) **###
2 vessel epicardial spasm	37 (25)	21 (25)	25 (33)
3 vessel epicardial spasm	39 (26)	8 (9) **	29 (39) ###
LVEF by UCG (%) mean ± SD	67 ± 9	68 ± 8	67 ± 7
Coronary risk factors			
Hypertension	68 (45)	27 (32) *	21 (28) *
Dyslipidemia	70 (47)	41 (48)	40 (53)
Diabetes mellitus	29 (19)	14 (16)	12 (16)
History of smoking	107 (71)	75 (88) **	60 (80)
Medications before ACh testing			
Calcium channel blocker	97 (65)	56 (66)	54 (72)
Nitrate or nicorandil	83 (55)	44 (52)	51 (68)
Beta blocker	8 (5)	4 (5)	3 (4)
ACEI or ARB	26 (17)	6 (7) *	12 (16)
Statin	41 (27)	5 (6) ***	20 (27) ###

(ACh: acetylcholine, RCA: right coronary artery, LCX: left circumflex artery, LAD: left anterior descending artery, LVEF: left ventricular ejection fraction, UCG: ultrasound cardiography, ARB: angiotensin receptor blocker, ACEI: angiotensin converting enzyme inhibitor, *: *p* < 0.05, **: *p* < 0.01, ***: *p* < 0.001 vs. diffuse spasm, ###: *p* < 0.001 vs. focal spasm).

**Table 4:** Comparisons of MACEs among five groups.

	Diffuse Spasm	Focal Spasm	Combined Spasm	Unclassified ACh Test	ACh Complete Negative
Readmission due to UAP	11 (7.3%) **	4 (4.7%)	2 (2.7%)	7 (3.8%)	1 (0.5%)
Sudden death	0	0	2 (2.7%)	0	0
ACS	0	1 (1.2%)	1 (1.3%)	1 (0.5%)	0
PCI	0	1 (1.2%)	0	0	0
CHF	0	0	1 (1.3%)	2 (1.1%)	0
CI	1 (0.7%)	0	0	1 (0.5%)	1 (0.5%)
**MACEs**	12 (8.0%) **	6 (7.1%) *	6 (8.0%) *	11 (5.9%) *	**2 (1.1%)**
Malignancy (death)	0	0	0	0	3 (1.6%)

(UAP: unstable angina pectoris, ACS: acute coronary syndrome, PCI: percutaneous coronary intervention, CHF: congestive heart failure, CI: cerebral infarction. MACE: major cardiac adverse event, ACh: acetylcholine, *: *p* < 0.05 and **: *p* < 0.01 vs. negative ACh test).

## Supplementary Materials

The following supporting information can be downloaded at: https://www.mdpi.com/article/10.3390/life12101465/s1, Supplementary Table S1: Univariable and multivariable analysis between positive spasm and negative result; Supplementary Table S2: Univariable and multivariable analysis between positive spasm and unclassified spasm.
