# Supplementary material for: Clinical Outcomes and Provoked Epicardial Spasm Phenotypes via Intracoronary Acetylcholine Testing in 680 Patients with Angina and Nonobstructive Coronary Arteries
Source: Life (Basel). 2022 Sep 21;12(10):1465. doi: 10.3390/life12101465 (PMC9605297; doi:10.3390/life12101465)
Supplement: Supplementary file 1 [file life-12-01465-s001.zip › life-1855700-supplementary.pdf]

Supplementary Table S1

Univariable and multivariable analysis between positive spasm and negative result

	Univariable analysis			Multivariable analysis		
	OR	95% CI	p value	OR	95% CI	p value
Age			0.869			
Sex	0.373	0.259-0.533	0.00000000987	0.732	0.395-1.360	0.321
Smoking	3.031	2.124-4.357	0.0000000000781	2.050	1.120-3.750	0.0198
Hypertension	0.899	0.650-1.243	0.526			
Dyslipidemia	1.341	0.975-1.845	0.0614	1.320	0.945-1.850	0.103
Diabetes mellitus	0.800	0.533-1.194	0.286			
LVEF by UCG			0.316			
Calcium channel blocker	3.009	2.173-4.182	0.00000000000314	1.880	1.280-2.770	0.00126
ACEI/ARB	1.031	0.650-1.630	0.912			
Nitrate or nicorandil	3.277	2.351-4.588	0.000000000000159	2.110	1.430-3.120	0.000174
Beta-blocker	0.598	0.292-1.179	0.120			
Statin	1.399	0.927-2.116	0.108			
Aspirin	1.835	1.150-2.953	0.00935	3.080	0.824-11.500	0.0946
Antiplatelet	1.619	1.042-2.529	0.025	0.366	0.104-1.290	0.118

(LVEF: left ventricular ejection fraction, UCG: ultrasonography, ACEI: angiotensin-converting enzyme inhibitor, ARB: angiotensin receptor blocker)

Supplementary Table S2

Univariable and multivariable analysis between positive spasm and unclassified spasm

	Univariable analysis			Multivariable analysis		
	OR	95% CI	p value	OR	95% CI	p value
Age			0.8329			
Sex	0.589	0.380-0.913	0.0132	0.761	0.391-1.48	0.423
Smoking	1.752	1.131-2.714	0.00946	1.350	0.703-2.590	0.367
Hypertension	0.978	0.660-1.453	0.924			
Dyslipidemia	1.091	0.743-1.605	0.707			
Diabetes mellitus	0.739	0.459-1.195	0.199			
LVEF by UCG			0.2529			
Calcium channel blocker	1.881	1.275-2.780	0.000883	1.380	0.893-2.120	0.148
ACEI/ARB	1.109	0.632-1.984	0.788			
Nitrate or nicorandil	2.251	1.523-3.347	0.000019	1.810	1.180-2.800	0.00709
Beta-blocker	0.809	0.338-1.995	0.678			
Statin	1.202	0.738-1.984	0.483			
Aspirin	1.484	0.857-2.638	0.159			
Antiplatelet	1.196	0.721-2.015	0.544			

(LVEF: left ventricular ejection fraction, UCG: ultrasonography, ACEI: angiotensin-converting enzyme inhibitor, ARB: angiotensin receptor blocker)
